# Prevalence of depression symptoms and associated sociodemographic and clinical correlates among Syrian refugees in Lebanon

**DOI:** 10.1186/s12889-021-10266-1

**Published:** 2021-01-26

**Authors:** Hady Naal, Dana Nabulsi, Nour El Arnaout, Lina Abdouni, Hani Dimassi, Ranime Harb, Shadi Saleh

**Affiliations:** 1 Global Health Institute, American University of Beirut, Beirut, Lebanon; 2 School of Pharmacy, Lebanese American University, Beirut, Lebanon; 3 Faculty of Health Sciences, American University of Beirut, Beirut, Lebanon

**Keywords:** Syrian refugees, Informal tented settlements, Sijilli, Mental health, Depression, Lebanon

## Abstract

**Background:**

Since the outbreak of the Syrian war in 2011, close to 6 million Syrian refugees have escaped to Syria’s neighbouring countries, including Lebanon. Evidence suggests rising levels of mental health disorders among Syrian refugee populations. Yet, to the best of our knowledge, large-scale studies addressing the mental health of adult Syrian refugees in Lebanon are lacking. We examined the prevalence of depression symptoms, which represent a common and debilitating mental health disorder among Syrian refugee populations in Lebanon, along with their sociodemographic and clinical correlates.

**Methods:**

A cross-sectional survey design was conducted as part of a collaborative project-“Sijilli”- led by the Global Health Institute at the American University of Beirut (Beirut, Lebanon) across 4 informal tented settlements for refugees (Beirut, Bekaa, North, South) in Lebanon among adult Syrian refugees (≥18), over a period extending from 2018 to 2020. The survey inquired about participants’ sociodemographic and clinical characteristics, and screened participants for symptoms of depression through sequential methodology using the Patient Health Questionnaire (PHQ-2 and PHQ-9).

**Results:**

A total of 3255 adult Syrian refugees were enrolled in the study. Of those refugees, 46.73% (*n* = 1521) screened positive on the PHQ-2 and were therefore eligible to complete the PHQ-9. In the entire sample (*n* = 3255), the prevalence of moderate to severe depression symptoms (PHQ-2 ≥ 2 and then PHQ-9 ≥ 10) was 22% (*n* = 706). Further analyses indicate that being ≥45 years of age (OR 1.61, 95% CI 1.13–2.30), a woman (OR 1.34, 95% CI 1.06–1.70), widowed (OR 2.88, 95% CI 1.31–6.32), reporting a neurological (OR 1.73, 95% CI 1.15–2.60) or a mental health condition (OR 3.98, 95% CI 1.76–8.97) are major risk factors for depression.

**Conclusion:**

Our study suggests that an estimated one in four Syrian refugees in Lebanon shows moderate to severe depression symptoms, and our findings have important public health and clinical implications on refugee health. There is a need to enhance screening efforts, to improve access and referral to mental health services, and to improve post-migration factors among Syrian refugees in Lebanon.

**Supplementary Information:**

The online version contains supplementary material available at 10.1186/s12889-021-10266-1.

## Background

The Syrian crisis has been widely described as one of the largest refugee crises of recent times [1, 2]. Close to 6 million Syrian refugees have fled to Syria’s neighbouring countries, namely Lebanon, Jordan, Turkey, Egypt, and Iraq [3]. Lebanon currently hosts over 1.5 million Syrian refugees, a number equivalent to 25% of its population [4], rendering it the country with the largest number of refugees per capita worldwide [5]. The massive influx of Syrian refugees to Lebanon, coupled with their increased demand for healthcare services, has significantly strained the country’s already fragile healthcare system, and has hindered its ability to cater to their health needs [6, 7].

Having escaped from conflict settings, refugees often experience a multitude of stressors such as traumatic events, multiple forms of losses, discrimination, and acculturation difficulties among others during their journey of displacement [1]. They have therefore significantly higher odds of developing mental health disorders compared to the general population [1]. Many refugees are survivors of exploitation, torture, and sexual and gender-based violence, which further exacerbate their vulnerability to health conditions [1]. That said, they are less likely to receive mental health services because of social stigma, language and cultural barriers, imbalanced power dynamics with service providers, limited access to services, and low mental health literacy, including lack of perceived need [1, 8–10]. Recent evidence indicates that mental health is one of the most pressing health needs among Syrian refugees in Lebanon and neighbouring countries [11].

Major Depression Disorder (MDD), Post-Traumatic Stress Disorder (PTSD), and other anxiety disorders have been reported as the most common mental health disorders among Syrian refugees [12, 13], and they tend to be comorbid conditions. In the general population, MDD in particular, is the third leading cause of years lived with disability (YLDs) [14], and is considered a strong risk factor for suicide [15, 16]. Therefore, depression warrants special attention among this population due to its long-term implications that may impair social, individual, and vocational functioning, factors that are essential for survival, productivity, and resettlement [17, 18]. In one meta-analysis that included 24,051 refugees from multiple nationalities pooled from international studies, 44% were found to have symptoms of depression [19]. Despite certain limitations of that review, such as heterogeneity of the included samples, this finding mirrors others in the literature addressing Syrian refugees in Arab settings [20–23]. Furthermore, previous reports from the literature assessing prevalence rates of depression among Syrian refugees in developed and developing countries have shown disparities in the findings. Depression prevalence rates were reportedly lower in developed countries compared to developing countries, which could be attributed to the limited capacities of the latter to cope with the needs of these vulnerable populations. In Germany, depression was detected in close to 14.5% of a Syrian refugee sample [13], as opposed to 37.4% in Turkey [12], and 43% in Lebanon [20].

Previous research suggests that despite the harsh conditions that Syrian refugees often experience, some key post-displacement variables may act as protective factors, may buffer the severity and incidence of mental health conditions, and may contribute to posttraumatic growth [13, 24]. For instance, obtaining a visa or residence permission, residing in acceptable living conditions, receiving financial support, and having access to social and healthcare services, among others, have contributed to better mental health outcomes [13]. Rightfully so, findings have confirmed that besides the existing acute and chronic stressors, the mental health of refugees largely depends on the social, economic, and cultural environments associated with their pre and post-displacement experiences [25]. However, the status of Syrian refugees in Lebanon is far from being ideal, with potential protective factors being compromised and minimally available [26]. As an example, Syrian refugees have restricted rights in Lebanon, which limit their access to proper healthcare, education, and employment opportunities, due to the lack of a clearly defined legal and administrative framework under which they can operate [26, 27]. Such systemic precariousness excludes potential opportunities for long-term integration, and places Syrian refugees in Lebanon at increased risk of developing mental health problems [28, 29]. In addition to that, Syrian refugees lack basic needs such as food, water, and shelter. Their acculturation is also compromised, as they tend to experience discrimination resulting from the strained Lebanese-Syrian relations due to host community fatigue with their protracted presence, largely due to job competition and exhaustion of resources and services [30]. This discrimination is also manifested as part of the larger socio-political climate in which continuous pressures are being exerted on Syrian refugees that threaten their physical, financial, and social security [26, 31, 32].

Finally, the lack of sustained funding for mental health services, the fragmented mental healthcare system, and the scarcity of research make it extremely difficult to understand and respond to the psychosocial needs of refugees. Syrian refugees, being a high-risk group, have unique psychosocial needs that should be clearly addressed by mental health workers, based on evidence-driven and culturally-sensitive findings. That said, concerted efforts have been made by the National Mental Health Program (NMHP) at the Ministry of Public Health (MoPH) in Lebanon in collaboration with other healthcare and humanitarian actors to address this problem [6, 7, 33, 34]. Since the inception of the Mental Health and Substance Use Strategy for Lebanon 2015–2020, the MoPH has worked on integrating mental health services into the primary healthcare centres (PHCs) by training healthcare workers on the Mental Health Gap Action Program (mhGAP) to enhance access to mental health care [35]. In addition, the MoPH established the Mental Health and Psychosocial Support Task Force (MHPSS-TF) in collaboration with the World Health Organization (WHO) and the UNICEF to coordinate the work of over 62 actors on the mental health and psychosocial support within the Syrian crisis response in Lebanon [7]. This has increased the efficiency and effectiveness of efforts targeting this problem.

In this context and despite the established initiatives, to our knowledge, there are limited large-scale studies on the prevalence of depression and its correlates among adult Syrian refugees in the Middle East. In fact, despite the urgency of the crisis, according to a recent systematic review, only six studies emanating from a conflict-affected low-to middle-income country were conducted in the Middle East to assess the prevalence rates of mental health disorders among refugees [36]. Clearly, there is an immense need for further research on the mental health of Syrian refugees in this region to better understand the associated risk factors, and to support the development and implementation of global mental health policies addressing this population [36].

The present study aims to examine the prevalence of depression symptoms and their sociodemographic and clinical correlates among Syrian refugees in Lebanon. In doing so, we provide researchers, policy makers, and practitioners with a comprehensive understanding of depression among migrating populations, which would constitute a foundational base for future interventions and related programs and policies.

## Methods

### Design & Population

Our study is a secondary analysis of de-identified data from the “Sijilli” (Electronic Health Records (EHR) for Refugees) database [37]. The Sijilli database includes data on 10,082 Syrian refugees in Lebanon, collected between July 2018 and January 2020 through primary field-based data collection conducted by the Global Health Institute at the American University of Beirut in partnership with Epic Systems Corporation. Data collection took place in different informal tented settlements for refugees across Lebanon, covering the Bekaa, North Lebanon, Beirut/Mount Lebanon, and South Lebanon areas. The sample size in each of these locations was proportionate to the overall Syrian refugee population residing in the latter based on UNHCR data [38] and includes 3565 refugee records (35.4%) from Bekaa, 2657 refugee records (26.4%) from North Lebanon, 2146 refugee records (21.3%) from Beirut, and 1714 refugee records (17.0%) from South Lebanon. The Sijilli database includes records of Syrian refugees of all ages, and covers 7 sections (see Additional File 1): socio-demographic information (e.g. age, gender, Syrian governorate of origin, location of the settlement, and year of migration to Lebanon), health behaviours (e.g smoking, alcohol drinking, and physical exercise), medical and surgical history, Obstetrics and Gynaecology (OBGYN) conditions, medication use, vaccination history, and mental health screening. The mental health screening was completed through the Patient Health Questionnaire-9 (PHQ-9). Any mental health disorder reported by refugees was noted right after PHQ-9 administration. In this study, we only analysed the data of adult Syrian refugees who were 18 years of age or above (*n* = 3255).

### Measures

From the collected data, we examined sociodemographic variables of age, gender, marital status, country of origin, time of arrival to Lebanon, location of settlement, and current occupation, and clinical variables of tobacco and alcohol use, medical conditions such as diabetes, cardiovascular diseases (CVD), neurological conditions, coronary artery diseases, use of psychiatric medication, and mental health conditions. Symptoms of depression were assessed in two phases using sequential methodology, which included administering the PHQ-2 followed by the PHQ-9 for those who screen positive on the former. This method has been widely used and is especially recommended for efficient data collection in conflict settings such as informal tented settlements [39].

The PHQ-9 includes 9 items rated on 4-point Likert ranging from 0 to 3, and yields a total score ranging from 0 to 27. The PHQ-9 score may be used as a categorical outcome of no, mild, moderate, moderately severe, and severe symptoms of depression, or may be used as a continuous score [40]. This measure screens for symptoms of depression experienced within the last 2 weeks and has previously shown evidence of validity, reliability, and unidimensionality [41]. In the present study, the PHQ-9 has shown very good reliability, with an alpha coefficient of 0.856.

### Analysis

All collected data were managed and analysed using SPSS v26. Descriptive statistics for sample characteristics were computed for adults using frequencies and percentages for categorical data. Internal consistency of the PHQ-9 was testing using Cronbach alpha. As recommended by a previous study [39], participants who screened positive on the PHQ-2 (received a score of 2 or above) were selected for further analysis in the regression model. PHQ-9 score was categorized into no symptoms (0–4), mild (5–9), moderate (10–14), moderately severe (15–19), and severe (20–27). Positive PHQ-9 was represented by a score of 10 or higher, as per previous studies in the literature [17]. PHQ-9 categories and average scores, as well as individual PHQ-9 items were summarized and reported as frequencies and percentages. Univariate and multiple logistic regression models were computed to determine the association of the PHQ-9 categories (mild and lower, score of 0–9; vs moderate to severe, score of 10 or higher) with the sample characteristics. Coefficients and standard errors were exponentiated to produce odds ratios and 95% confidence intervals. All analyses were run at the 0.05 statistical significance level.

## Results

### Participants

A total of *n* = 3255 adult Syrian refugees were included in this study, and their characteristics are presented in Table 1. The mean age was 36.53 with a standard deviation of 13.87. Most of the participants were women (67.1%), married (78.4%), had arrived to Lebanon between 2012 and 2015 (62.6%), originating from Hama governorate in Syria (18.9%), currently residing in the Bekaa governorate in Lebanon (32.6%), and unemployed (74.6%). The majority of participants reported never using tobacco (67.6%) and alcohol (99.5%), and only 1.2% reported using psychiatric medication such as antidepressants, antipsychotics, and anxiolytics. The most commonly reported medical condition was hypertension (10%), and the presence of any mental health condition was reported among only 2.1% of the study population.

**Table 1:** Characteristics of the present sample (*N* = 3255)

	Total	
	N (%)	
**Gender**		
Female	2184	67.1%
Male	1071	32.9%
**Age (mean & SD)**	36.53 (13.87)	
18–25	825	25.3%
26–35	878	27.0%
36–45	772	23.7%
> 45	780	24.0%
**Marital Status**		
Married	2553	78.4%
Single	552	17.0%
Divorced	32	1.0%
Widowed	106	3.3%
Separated	12	0.4%
**City of Origin**		
Hama	616	18.9%
Aleppo	606	18.6%
Raqqa	515	15.8%
Idlib	498	15.3%
Homs	456	14%
Deir ez-Zor	299	9.2%
Others	262	8.0%
**Time of Arrival to Lebanon**		
< 2011	616	18.9%
2012–2015	2039	62.6%
2016–2020	600	18.4%
**Location**		
Beirut	670	20.6%
Bekaa	1062	32.6%
North Lebanon	942	28.9%
South Lebanon	581	17.8%
**Current Occupation**		
Unemployed	2420	74.6%
Employed	826	25.4%
**Exercise**		
Yes	611	20.80%
No	2328	79.20%
**Tobacco Use**		
No use	2109	67.6%
Ex-user	91	2.9%
Current	920	29.5%
Hookah	171	5.30%
Cigar	46	1.40%
Cigarettes	797	24.50%
**Alcohol Use**		
Yes	17	0.50%
No	2992	99.50%
**Medication Use (Psychiatric)**		
Antidepressant	28	0.9%
Antipsychotic	9	0.3%
Anxiolytic	7	0.2%
**Medical Conditions**		
Hypertension	326	10.0%
Neurological disorders	196	6.0%
Diabetes	185	5.7%
Cardiovascular Disease	175	5.4%
Coronary Artery Disease	62	1.9%
**Mental Health Conditions**		
Any Mental Health Condition	40	1.23%

### Prevalence and distribution of depression symptoms

**Fig. 1:**
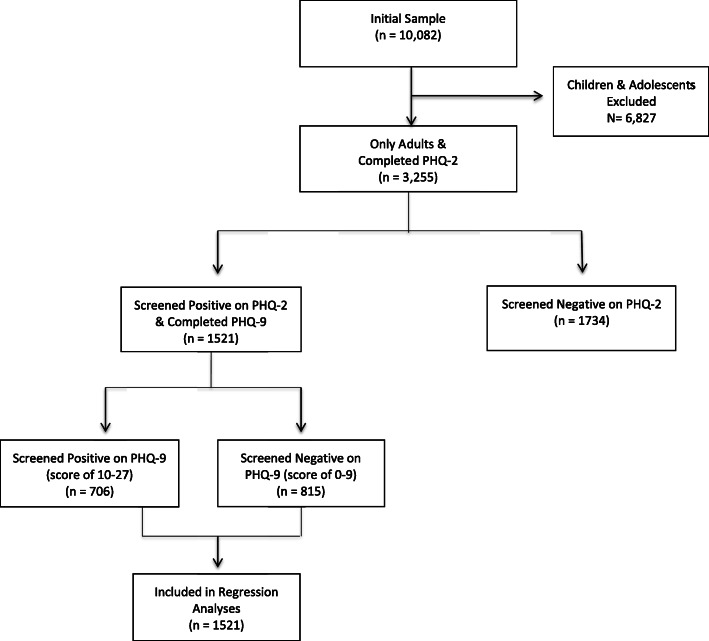
Flowchart for participant selection. This flowchart showcases the process by which recruited participants were selected from inclusion in the study analysis

Of those *n* = 3255 adults, almost a quarter (*n* = 706) screened positive on the PHQ-2 (scored ≥2) and then on the PHQ-9 (scored ≥10), indicating a 22% prevalence of moderate to severe depression symptoms in the total population (n = 3255). Less than half of the sample 46.7% (*n* = 1521) completed the full PHQ-9 and were subsequently included in the regression analysis. The remaining half (*n* = 1734) were excluded from the regression analysis due to screening negative on the PHQ-2 (see Fig. 1). More than half (53.6%) of those who completed the PHQ-9 showed no or mild depression symptom severity, however, the rest (46.4%) showed moderate to severe symptom severity (see Table 2). The highest reported symptom severity was for “*feeling little interest and pleasure in doing things that used to be enjoyable”*, “*feeling down and depressed on most days”*, and “*having little energy to complete daily tasks”*. Whereas the least reported symptoms were for “*moving or speaking very slowly”, and* “*having suicidal thoughts”*.

**Table 2:** Distribution of depression symptoms *N* = 1521

	N	%
**Depression Symptoms**		
No symptoms (0–4)	201	13.2
Mild (5–9)	614	40.4
Moderate (10–14)	372	24.5
Moderately Severe e(15–19)	210	13.8
Severe (20–27)	124	8.2
Mean (SD)	10.4 (5.7)	
**Depression Screening**		
Mild and lower (0–9)	815	53.6
Moderate to Severe (10–27)	706	46.4
**PHQ1: Interest/pleasure**		
Not at all	38	2.5
Several days	796	52.3
More than half of days	395	26.0
Nearly everyday	285	18.7
**PHQ2: Down, depressed**		
Not at all	14	.9
Several days	722	47.5
More than half of days	444	29.2
Nearly everyday	339	22.3
**PHQ3: Sleep**		
Not at all	398	26.2
Several days	546	35.9
More than half of days	350	23.0
Nearly everyday	216	14.2
**PHQ4: Energy**		
Not at all	191	12.6
Several days	638	41.9
More than half of days	429	28.2
Nearly everyday	255	16.8
**PHQ5: Appetite**		
Not at all	628	41.3
Several days	472	31.0
More than half of days	254	16.7
Nearly everyday	163	10.7
**PHQ6: Feeling bad/failure**		
Not at all	557	36.6
Several days	544	35.8
More than half of days	249	16.4
Nearly everyday	157	10.3
**PHQ7: Concentrating**		
Not at all	601	39.5
Several days	477	31.4
More than half of days	292	19.2
Nearly everyday	145	9.5
**PHQ8: Move/speak slowly**		
Not at all	805	52.9
Several days	422	27.7
More than half of days	199	13.1
Nearly everyday	81	5.3
**PHQ9: Better off dead**		
Not at all	932	61.3
Several days	320	21.0
More than half of days	155	10.2
Nearly everyday	104	6.8
**PHQ10 Effect work/people**		
Not difficult at all	485	31.9
Somewhat difficult	621	40.8
Very difficult	255	16.8
Extremely difficult	124	8.2

### Sociodemographic and clinical correlates of depression symptoms

Based on the results of the first regression model at the bivariate level, being over 45 (OR 1.74, 95% CI 1.25–2.14), a woman (OR 1.38, 95% CI 1.00–1.74), divorced/separated (OR 3.27, 95% CI 1.51–7.08), on psychiatric medication (OR 2.73, 95% CI 1.04–7.14), having hypertension (OR 1.36, 95% CI 1.00–1.84) or any nervous-system related disorder (OR 1.76, 95% CI 1.78–2.63), or mental health condition (OR 3.70, 95% CI 1.67–8.26) significantly increased the odds for showing more severe symptoms of depression (see Table 3). On the other hand, informal tented settlement location, current occupation, tobacco and alcohol use, and having diabetes or CVDs showed no statistically significant association with symptoms of depression.

**Table 3:** Sociodemographic and clinical correlates of depression symptoms at the bivariate level (*n* = 1521)

	N	%	*p*-value	Odds ratio	Lower L	Upper L	ROC area (95%CI)
**Age**							
18–25 (ref)	89	39.0%		Ref			
26–35	197	46.9%		1.380	.994	1.915	
36–45	190	43.5%		1.201	.867	1.665	
> 45	230	52.8%	.004	1.744	1.259	2.415	.55 (.52–.58)
**Gender**							
Male	169	40.6%		ref			
Female	537	48.6%	.006	1.382	1.100	1.736	.53 (.50–.56)
**Marital Status**							
Married	556	44.9%		ref			
Single	85	47.5%		1.108	.809	1.516	
Widowed	41	56.9%		1.620	1.003	2.617	
Divorced/separated	24	68.6%	<.001	3.266	1.506	7.084	.53 (.50–.56)
**Period of Stay** (continuous)			.947	1.002	.954	1.051	.50 (.47–.53)
**Location**							
Beirut	128	43.0%		ref			
North	187	46.3%		1.145	.847	1.547	
South	94	46.3%		1.145	.800	1.640	
Bekaa	297	48.2%	.524	1.237	.936	1.634	.52 (.49–.55)
**Current Occupation**							
Employed	161	44.6%		ref			
Not Employed	542	47.0%	.431	.909	.717	1.153	.51 (.48–.54)
**Tobacco Use**							
Never User	455	46.6%		ref			
Current	234	46.9%	.906	1.013	.816	1.257	.50 (.47–.53)
**Alcohol Use**							
No	675	47.3%		ref			
Yes	3	42.9%	.814	.836	.186	3.747	.50 (.47–.53)
**Psychiatry Medication Use**							
No	692	46.1%		ref			
Yes	14	70.0%	.033	2.728	1.043	7.137	.51 (.48–.54)
**Medical Conditions**							
Diabetes No	648	45.9%					
Diabetes Yes	58	53.7%	.115	1.369	.925	2.028	.51 (.48–.54)
Hypertensive No	604	45.4%					
Hypertensive Yes	102	53.1%	.046	1.360	1.004	1.842	.52 (.49–.55)
Cardiovascular Disease No	656	45.9%					
Cardiovascular Disease Yes	50	53.8%	.143	1.368	.898	2.084	.51 (.48–.54)
Nervous Disorder No	643	45.4%					
Nervous Disorder Yes	63	59.4%	.005	1.759	1.177	2.628	.52 (.49–.55)
Mental Health No	681	45.8%					
Mental Health Yes	25	75.8%	<.001	3.703	1.660	8.263	.51 (.48–.54)

Note: the dependent variable is the dichotomized PHQ-9 (mild and lower VS moderate to severe)

At the multivariable level of analysis, comparable results were obtained, whereby being ≥45 of age (OR 1.61, 95% CI 1.13–2.30), a woman (OR 1.34, 95% CI 1.06–1.70), widowed (OR 2.88, 95% CI 1.31–6.32), reporting a neurological disorder (OR 1.73, 95% CI 1.15–2.60) or a mental health condition (OR 3.98, 95% CI 1.76–8.97) significantly increased the odds of screening positive for moderate to severe depression symptoms (see Table 4). However, unlike the bivariate model, being on psychiatric medication and presenting with hypertension were not significantly associated with the increased odds of depression severity symptoms.

**Table 4:** Sociodemographic and clinical correlates of depression symptoms at the multivariable level (*N* = 1521)

Risk factor	B	SE	*p*-value	Odds ratio	Lower L	Upper L	ROC (95% CI)
**Multivariable Logistic Regression Model**							.60 (.57–.63)
**Age groups**							
18–25 (ref)							
26–35	.316	.172	.066	1.371	.979	1.921	
36–45	.162	.172	.345	1.176	.840	1.648	
> 45	.476	.181	.009	1.610	1.128	2.296	
**Gender**							
Male (ref)							
Female	.293	.121	.015	1.340	1.058	1.698	
**Marital Status**							
Married (ref)							
Single	.194	.166	.243	1.214	.877	1.681	
Widowed	.299	.254	.008	2.883	1.315	6.321	
Divorced/Separated	1.059	.400	.240	1.349	.819	2.221	
**Medical Conditions**							
Nervous Disorder No							
Nervous Disorder Yes	.550	.208	.008	1.732	1.153	2.604	
Mental Health No							
Mental Health Yes	1.380	.415	.001	3.976	1.762	8.973	
Hypertension	.145	.168	.387	1.156	.832	1.608	
Psychiatric Medication	.496	.517	.337	1.643	.596	4.527	

Note: the dependent variable is the dichotomized PHQ-9 (mild and lower VS moderate to severe)

## Discussion

In the present study, we report the prevalence of depression symptoms, which represent one of the most common and debilitating mental health disorders among Syrian refugees, and we explore their sociodemographic and clinical correlates. The main strength of this study is that to our knowledge, it is one of the very few regional, and the only national large-scale study addressing this issue among a representative sample of adult Syrian refugees in Lebanon.

As expected, the prevalence of depression symptoms among the study population was high, with an estimated one in four refugees meeting criteria for moderate to severe depression symptoms. Positive PHQ-9 screening and consequently, moderate to severe depression symptoms, was detected in 25% of the study population, which is considerably higher than depression rates (9.9%) previously reported among the general population in Lebanon [42]. Our findings contrast a previous study that used the same depression measure on Syrian refugees in Germany, and in which only 14% of their sample screened positive for moderate to severe depression symptoms [13]. One of the main findings of that study was that post-migration conditions and future positive prospects in host countries may be protective against mental health disorders among these populations [13]. Indeed, this could explain the discrepancy between their findings and ours, considering that post-migration factors in Lebanon are poor and may ultimately present important risk factors instead of being protective against mental health disorders [26].

In Lebanon, many post-migration variables present important obstacles towards adequate health and survival. For example, the high prevalence of depression symptoms could be attributed to numerous external factors beyond their exposure to traumatic events, such as the difficult conditions in which Syrian refugees live, the limited opportunities for development, and the many challenges associated with their social integration and acculturation [26]. Additionally, the constant internal and regional socioeconomic and political conflicts promote little hope for refugees to settle in a stable context unless they travel to more developed countries, which is a solution Syrian refugees commonly request to overcome their documented adverse living conditions [26]. The existing economic difficulties in Lebanon, which are now exponentially compounded by the fall of Lebanese pound [43], and by the colossal explosion that devastated the capital Beirut in August 2020 [44], may place Syrian refugees under further instability and vulnerability. With that said, the situation is currently expected to be worse in terms of mental health outcomes, considering the COVID-19 pandemic, which has restricted mobility, tremendously challenged the attainment of basic survival needs, induced added stress, and further limited opportunities for work and social interactions [45].

In response to the Syrian crisis, the MoPH in collaboration with the Ministry of Social Affairs and with local and international non-governmental organizations (NGOs) have been providing free-of-charge primary healthcare services, including mental health services, for UNHCR-registered Syrian refugees. Yet, due to limited financial capacities, these efforts have been reportedly unable to meet the increasing needs of these vulnerable populations [46, 47]. The situation is even worse for unregistered refugees who have restricted capacities to receive the appropriate healthcare support [47]. Despite these efforts, symptoms of depression remain high, as indicated by our findings. Although mental health services and psychosocial interventions may induce relief of depression severity over the short-term, long-term improvements may require complementary macro-level changes in the living conditions of refugees, their legal status, and the need to foster positive future prospects.

It is also possible that mental health services are not reaching enough refugees. Notwithstanding the value of the provided services, Syrian refugees cite many barriers to seeking mental health services in Lebanon, including lack of trust in and limited knowledge of available services, limited mental health literacy and perceived need for treatment, lack of services especially in rural areas, associated difficulties in commuting, financial barriers and lack of mental health coverage, and social stigma which may impede refugees seeking healthcare fearing of shame and discrimination [46]. Furthermore, due to pervasive cultural beliefs, Syrian refugees tend to seek religious healers as a first line of treatment for mental illness given their perceived cultural appropriateness and their reduced association with social stigma when compared to mental health professionals [48].

Despite high symptoms of depression in our sample, our findings are favourable in comparison to the last study that evaluated the prevalence of depression among adult Syrian refugees in Lebanon 5 years ago, in which a depression prevalence rate of 43.9% was reported [20]. Although some important limitations may prohibit this comparison, such as their reliance on a clinical diagnosis as opposed to using a screening instrument, and their smaller sample size (*n* = 310) [20], our findings may point towards an overall improvement. However, this could also be a result of the different sample characteristics, considering that Naja et al. [20] study represented refugees seeking social and healthcare services from two non-governmental organizations, whereas ours included a randomly selected representative sample of participants from the general population of Syrian refugees across Lebanon; under these different contexts, our sample would be expected to score lower on depression.

In terms of its correlates, our findings show that age, gender, and marital status are strongly associated with an increased risk for depression. We found that older age is associated with higher risk of depression, which contrasts previous findings showing an inverse relationship [13], or no correlation [17, 20] between these variables. In specific, individuals over 45 years of age are at highest risk of developing depression, and this may be a result of several contributing factors. From a social perspective, older individuals in the Arab region are regarded as pillars of their families, and they tend to hold leadership roles in their communities [49, 50]. Previous research suggests that as a result of the war, this community role may be disrupted, impacting familial connectedness and social ties, and bringing along feelings of isolation and inadequacy [49]. From a clinical perspective, older individuals often have pre-existing and chronic conditions which may warrant further medical attention. In this regard, unmet healthcare needs due to the limited resources in these communities may further aggravate their mental health conditions and general well-being. Finally, comorbidities between cognitive disorders and depression may be more pronounced and severe among this age group, which may impact their well-being and overall functionality [51].

In terms of gender differences, previous research consistently reported higher prevalence rates of depression among women compared to men across studies among the general population, and this is also true of research among Syrian refugees [13, 17] congruent with our findings. Although some of the same justifications that have been previously cited in the literature may still be used to explain these gender-based variations, other explanations that are specific to Syrian refugees in Lebanon should be considered. For example, women in refugee settings tend to be subjected to early and forced marriage and bear family responsibilities at an early age, in addition to being exposed to sexual harassment and violence in the household and community at large [46], all of which are potential stressors that increase the risk of developing moderate to severe depression symptoms. Also, these women allude to child rearing and associated responsibilities as important sources of stress and anxiety, especially when considering their worries about the discrimination and bullying their children may face in schools in Lebanon [46].

The association between marital status and depression has been previously examined among Syrian refugees, whereby being married was found to be protective against depression in one study, potentially due to its association with social support [17], and where no relationship was found in others [12, 13, 20]. In our study, being widowed increased the odds of having depression, but this was not the case for being single or divorced. The experience of going through a death of a spouse in this community could place individuals at higher risk of developing depression than if they were single or divorced.

On the other hand, several variables were found not to be related to depression, most importantly the location of the informal tented settlement and the period of stay in Lebanon. It is possible that the properties of the four examined informal tented settlements for refugees in Lebanon entail similar living conditions that are below the appropriate standards and share similarity in terms of the availability of support and healthcare services, which translate into poor mental health among their residents. As for the period of stay in Lebanon, although previous studies suggested that a longer period of stay is associated with higher risk of depression [17], in our study we did not find any such association. This suggests that the period of stay in Lebanon is not correlated with the risk of developing depression, given that the mental health of Syrian refugees may be equally compromised among newcomers and long-term residents. That said, arriving in Lebanon before and after the breakout of the Syrian war in 2011 was accounted for in the study analysis, and no significant differences in depression scores were observed.

In terms of the clinical characteristics, we found that reporting a neurological condition or a history of mental illness increased the risk of depression, presumably because mental illness is directly related to depression symptoms, and neurological conditions are frequently comorbid with depression [52]. On the other hand, diabetes, CVDs and coronary artery diseases were not found to be risk factors for depression, although these have been associated with depression in previous studies [53, 54]. Similarly, although reporting hypertension and the use of psychiatric medication have shown a statistically significant association with depression at the bivariate level of analysis, this was not replicated at the multivariable level. This means that unlike neurological conditions, non-communicable diseases may not be significant risk factors for developing depression among Syrian refugees in Lebanon. Possibly, these associations might be clouded by the overall high symptoms of depression in the study population.

In order to gain a clearer understanding of mental health disorders in this population, we recommend for future research to explore a wider range of disorders in this population such as anxiety disorders, PTSD, and substance use disorders for example to better understand their prevalence and correlates. Also, large-scale studies are needed to examine the relationship between pre and post migration factors such as legal status, living conditions, and available aids and services on the mental health of this population in this area.

## Recommendations & implications

Our findings indicate that the prevalence of depression symptoms among Syrian refugees in Lebanon is high. In light of this, an increase in screening efforts and referral mechanisms to PHCs and other health facilities is highly needed to improve access to mental health services and to reduce depression symptoms. One recommended way to do that is through task shifting, by conducting more capacity building initiatives that train non-specialized community health workers to deliver basic mental health services to their communities and to conduct referrals [55, 56]. According to a recent national facility assessment, almost 32% of PHCs in Lebanon are currently delivering mental health services following the mhGAP training, and almost 50% of them cover rural areas [57], where most refugees reside. The integration of telemental health services into PHCs, community-based organizations, and other healthcare facilities that could be accessed by refugees may also be a suitable option to enhance the reach of these services [58, 59]. Telemental health services have strong potential in overcoming traditional barriers to mental health service use such as transportation challenges, unequal distribution of specialists among others, and may enhance access to services [58]. Importantly, considering the mobility restrictions brought forth by the COVID-19, telemental health adheres to the social distancing requirements, and enables access to mental health services for remote and underserved populations [45]. PHCs in Lebanon are usually equipped with the minimally required technological hardware and software needed for that, they tend to have minimally available specialized mental health care services, and their catchment areas tend to reach rural and underserved populations [57]. To that end, special attention should be given to high-risk groups such as older adults, women, and widowed individuals, particularly those with a history of neurological or mental health conditions. More importantly, using a culturally-sensitive approach, efforts to combat social stigma and improve mental health literacy in these communities are necessary to encourage treatment seeking when available. For example, one recent qualitative study suggested that collaborations between mental health professionals and community religious healers may be a key factor to creating pathways for referrals to mental health services [48]. Additionally, it is also important to provide minimally required and essential resources for survival, in parallel as a complement to the existing mental health services. In this regard, different aids that improve the living conditions and the quality of life for Syrian refugees are crucial, especially those that have been consistently identified as key protective factors. For example, providing options to 1) improve the financial situation in order for them to be able to meet their basic survival needs, 2) facilitate interactions with host communities and reduce tensions, and 3) improve the legal status of refugees, which help them benefit from the available services.

## Limitations

Some limitations should be considered in light of our findings. First, it is important to note that the study population is not entirely homogeneous because it included individuals who came before and after the breakout of the Syrian war in 2011, which implies different levels of exposure to war trauma. Nevertheless, we accounted for this in our analysis by comparing depression symptoms between both groups whereby no significant differences in symptom severity were observed. Second, biases such as social desirability may have influenced the collected data (e.g. symptoms of depression, alcohol use, employment etc.) since participants were administered the questionnaire through in-person interviews rather than by self-reported means, given the high levels of reported illiteracy in informal tented settlements. Third, pre-and post- migration data such as legal status, household and living conditions, and the quality of healthcare provision among others were not collected, which could otherwise have provided a clearer association between migration status and depression. Lastly, reliance on self-reported data rather than official diagnostic reports may have resulted in under- or overestimation of the reported health conditions.

## Conclusion

Mental health is of growing importance in refugee populations due to the increased vulnerability of these populations to mental health disorders. Our study findings revealed high prevalence of depression symptoms among the study population, with one in four refugees meeting criteria for moderate to severe depression symptoms. Furthermore, we found an association between potential risk factors such as, being of older age, a woman, widowed, having a neurological condition or a history of mental illness and the increased odds of having depression symptoms. Our findings bear important public health and clinical implications on refugee health, and call for the enhancement of screening efforts, the need to improve access and referral to mental health services, and the importance of improving post-migration factors such as those related to living conditions, acculturation, and legal status.

## Supplementary Information

**Additional file 1.** This questionnaire showcases the variables used to collect data through the Sijilli Database.

## Abbreviations

AUB: American University of Beirut
COVID-19: Corona virus diseases – 19
CVD: Cardiovascular disease
GHI: Global Health Institute
IRB: Institutional Review Board
MDD: Major Depression Disorder
mhGAP: Mental Health Gap Action Program
MHPSS-TF: Mental Health and Psycho Social Support – Task Force
MoPH: Ministry of Public Health
NMHP: National Mental Health Program
OBGYN: Obstetrics and Gynaecology
PHC: Primary Healthcare Center
PHQ-9: Patient Health Questionnaire – 9
PTSD: Post traumatic stress disorder
WHO: World Health Organization
