# Supplementary material for: Prevalence of depression symptoms and associated sociodemographic and clinical correlates among Syrian refugees in Lebanon
Source: BMC Public Health. 2021 Jan 26;21:217. doi: 10.1186/s12889-021-10266-1 (PMC7836044; doi:10.1186/s12889-021-10266-1)
Supplement: Supplementary file 1 — Additional file 1. This questionnaire showcases the variables used to collect data through the Sijilli Database. [file 12889_2021_10266_MOESM1_ESM.pdf]

1. Personal information
- First name
- Father's name
- Last name (if female indicate maiden name)
- Mother's name
- Date of birth
- Past & current occupation
- Marital status
- Consanguinity state of parents
- Allergy
- Blood type
- Sex
- City of origin

2. Social & Lifestyle
- Smoking status (if smoker indicate packs per year)
- Alcohol intake (if regular drinker indicate frequency cups per week)
- Exercise (if yes, indicate type & frequency per week)

3. Medical Conditions
- Medical conditions (for each specify year of diagnosis)

4. Surgical History
- Surgeries done (indicate for each date of surgery)

Institutional Review Board
American University of Beirut
28 FEB 2018
RECEIVED

Institutional Review Board
American University of Beirut
26 MAR 2018
APPROVED

Institutional Review Board
American University of Beirut

28 FEB 2018

RECEIVED

5. Mental Health assessment

Table 3. Patient Health Questionnaire-2: Screening Instrument for Depression

Over the past two weeks, how often have you been bothered by any of the following problems?	*Not at all*	*Several days*	*More than one-half the days*	*Nearly every day*
Little interest or pleasure in doing things	0	1	2	3
Feeling down, depressed, or hopeless	0	1	2	3

NOTE: *If the patient has a positive response to either question, consider administering the Patient Health Questionnaire-9 or asking the patient more questions about possible depression. For older adults, consider the Patient Health Questionnaire-9 or the 15-item Geriatric Depression Scale. A negative response to both questions is considered a negative result for depression.*

Adapted from patient health questionnaire (PHQ) screeners. http://www.phqscreeners.com. Accessed September 6, 2011.

Table 4. Patient Health Questionnaire-9: Screening Instrument for Depression

Over the past two weeks, how often have you been bothered by any of the following problems?	*Not at all*	*Several days*	*More than one-half the days*	*Nearly every day*
Little interest or pleasure in doing things	0	1	2	3
Feeling down, depressed, or hopeless	0	1	2	3
Trouble falling or staying asleep, or sleeping too much	0	1	2	3
Feeling tired or having little energy	0	1	2	3
Poor appetite or overeating	0	1	2	3
Feeling bad about yourself—or that you are a failure or have let yourself or your family down	0	1	2	3
Trouble concentrating on things, such as reading the newspaper or watching television	0	1	2	3
Moving or speaking so slowly that other people could have noticed. Or the opposite—being so fidgety or restless that you have been moving around a lot more than usual	0	1	2	3
Thoughts that you would be better off dead, or of hurting yourself in some way	0	1	2	3
	**Total:**	____ +	____ +	____

Interpretation

Total score	*Depression severity*
1 to 4	Minimal
5 to 9	Mild
10 to 14	Moderate
15 to 19	Moderately severe
20 to 27	Severe

Adapted from patient health questionnaire (PHQ) screeners. http://www.phqscreeners.com. Accessed September 6, 2011.

Institutional Review Board
American University of Beirut

26 MAR 2018

APPROVED

Institutional Review Board
American University of Beirut
28 FEB 2018
RECEIVED

6. Medication List
   - Generic Medication (for each indicate dosage, unit, frequency & date started)

7. Vaccine List
   - Tetanus-Diphtheria-Pertusis
   - Measles-Mumps-Rubella
   - Hepatitis B
   - IVP
   - Polio
   - OPV
   - Pneumcoccal
   - Haemphilus
   - BCG
   - Tuberculosis screening done (if yes indicate if positive or negative and whether a chest X-ray was performed or not)

   (Indicate for each if the vaccine is taken or not. If taken indicate date)

8. Comments

Institutional Review Board
American University of Beirut
26 MAR 2018
APPROVED
